# The behaviour of sea snakes (*Emydocephalus annulatus*) shifts with the tides

**DOI:** 10.1038/s41598-020-68342-2

**Published:** 2020-07-09

**Authors:** Claire Goiran, Gregory P. Brown, Richard Shine

**Affiliations:** 1 LabEx Corail and ISEA, Université de La Nouvelle-Calédonie, BP R4, 98851 Nouméa Cedex, New Caledonia; 2 Department of Biological Sciences, Macquarie University, Sydney, NSW 2109 Australia

**Keywords:** Ecology, Zoology

## Abstract

Tidal cycles are known to affect the ecology of many marine animals, but logistical obstacles have discouraged behavioural studies on sea snakes in the wild. Here, we analyse a large dataset (1,445 observations of 126 individuals) to explore tidally-driven shifts in the behaviour of free-ranging turtle-headed sea snakes (*Emydocephalus annulatus*, Hydrophiinae) in the Baie des Citrons, New Caledonia. Snakes tended to move into newly-inundated areas with the rising tide, and became more active (e.g. switched from inactivity to mate-searching and courting) as water levels rose. However, the relative use of alternative habitat types was largely unaffected by tidal phase.

## Introduction

Evolutionary shifts between terrestrial and marine habitats have occurred in several lineages of vertebrates, notably in mammals, reptiles and birds^1,2^. Such a habitat shift exposes organisms to novel abiotic and biotic challenges^3^; for example, oceanic habitats are more stable thermally than are many terrestrial habitats, but experience profound shifts in current and water depth with the tidal cycle^4,5^. In turn, tide-determined changes in biologically important processes (such as reproductive opportunities, prey availability and vulnerability to predators) have favoured adaptive matching of behaviour to tidal phase in many marine species^6–11^. Especially for organisms that inhabit relatively shallow waters, tidal cycles may be a key driver of habitat use, activity level and ecological interactions^4^.

Sea snakes have attracted little research in this respect, in contrast to the extensive information available on effects of environmental parameters on activity patterns of terrestrial snakes^12–14^. Logistical impediments to observing snakes underwater are responsible for that lack of information. Recent developments in telemetry are providing far more detailed datasets on activity patterns and habitat use of marine snakes^15–20^ but without direct information on behaviour. One striking exception involves studies showing that rising tides induce sea snakes (*Hydrophis elegans* and *H. major*) to shift habitat use (from open sites into seagrass beds) to avoid predation risk from sharks as water levels rise^21,22^. To our knowledge, the only other published study of effects of tidal cycles on sea snakes involved an amphibious species (*Laticauda colubrina*) that was observed on land, revealing a trend for movements between land and sea to be synchronised with high tides^23^. Information on the impacts of tidal cycles on sea snake behaviour (e.g., microhabitat use, activity levels) not only can clarify the natural history of these poorly-known animals, but also may help us to understand the proximate effects of anthropogenic challenges (e.g., coral bleaching) on populations of marine snakes. We exploited an unusual opportunity—an abundant population of individually-marked sea snakes, in an easily-accessible site—to gather a large dataset on the effects of tidal cycles on habitat use and activity patterns of these snakes. Given the shallow-water habitats in which our study species is found, we predicted that tidal cycles would influence the kinds of habitats in which snakes were found, whether or not they were active, and what kinds of activity would be exhibited.

## Results

We obtained data on 1,445 sightings of 126 individuals (1–39 sightings per snake). The sample size of sightings was slightly male-biased (57% vs. 43%) as was the overall sex ratio (68 males, 57 females). Reflecting ease of sampling, we gathered more data at high tide (50% of sightings) than at medium (28%) or low (22%) tides. Snakes were sighted most often while they were foraging (54% of observations) or inactive (34%), and less often while they were mate-searching and courting (10%) or being courted (2%).

### Effect of tide on water depth

**Figure 1:**
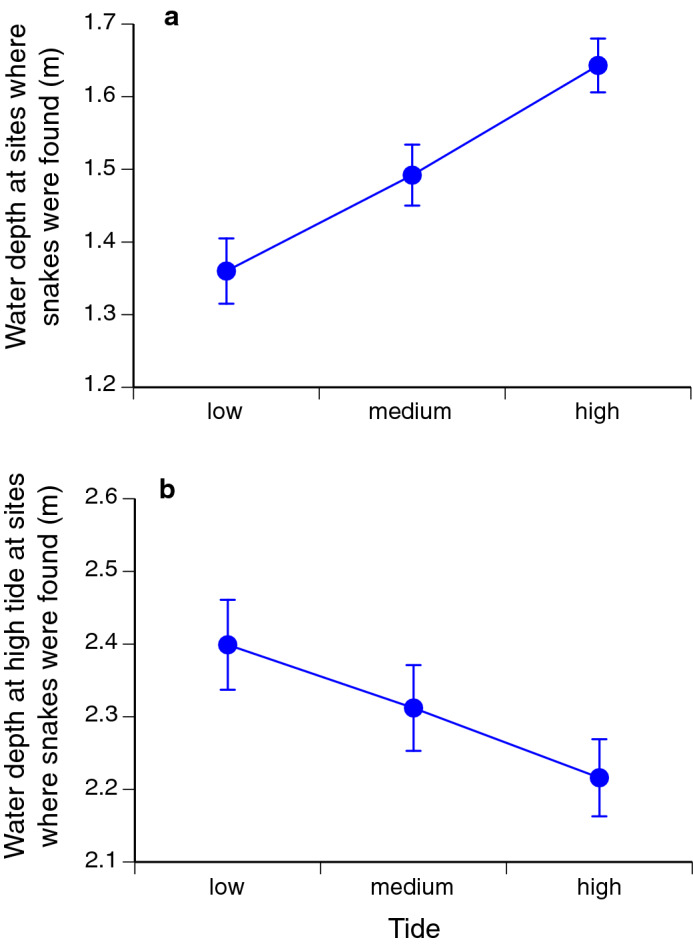
Effect of tidal phase on (**a**) water depth at time of capture in sites where we found sea snakes (*Emydocephalus annulatus*), and on (**b**) the depth of water at high tide at those same sites. Graphs show mean values (± 1 s.e.m.).

Tidal phase (low vs. medium vs. high) affected both the depth of water in which snakes were found (*F*_2,1369_ = 25.07, *P* < 0.0001; Fig. 1a) and the water depth at high tide at sites where snakes were found (*F*_2,1362_ = 6.29, *P* < 0.002; Fig. 1b). However, the two patterns were in opposite directions (Fig. 1). Snakes were generally in shallower water when found at a lower tide, as expected from the general reduction in sea level at low tide (Fig. 1a). In contrast, sites where we found snakes at high tide tended to be locations close to shore, that were not deeply inundated even at high tide; whereas sites where we found snakes at low tide tended to be further from shore, in locations that were deeply inundated at high tide (Fig. 1b). That is, snakes preferred shallow water regardless of the tide. That pattern could be due to snake behaviour (i.e. snakes move towards the shoreline at high tide) and/or to the difficulty of surveying for snakes in very shallow water. Both explanations may be true. The strongest evidence for shoreward migration of snakes at high tide comes from 34 records of snakes in quadrats that were completely dry at low tide. Any snakes remaining in those quadrats would be extremely obvious, yet we have never seen any in 2 decades of research on this system.

### Effect of tide on habitat use

**Figure 2:**
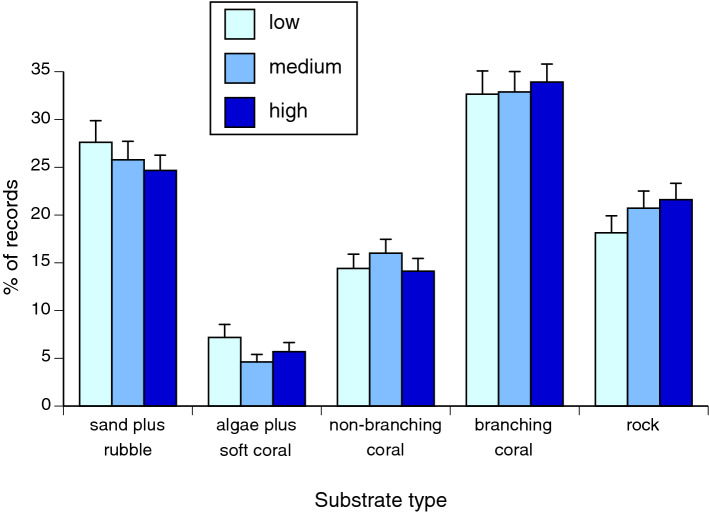
Effect of tidal phase on the habitat (substrate) types in sites where we found sea snakes (*Emydocephalus annulatus*) in the Baie des Citrons, New Caledonia. Bars show SEs.

Tide had no significant effect on snake associations with four of the five habitat characteristics (percent sand plus coral rubble, branching coral, non-branching coral, algae plus soft coral; all *F* < 2.68, all *P* > 0.069; Table 1, Fig. 2). However, snakes were more likely to be associated with rocky habitats during high or medium tides than during low tides (Fig. 2).

**Table 1:** Results of mixed model analyses on the effects of tide on characteristics of habitat used by sea snakes (*Emydocephalus annulatus*).

Response variable	Effect of tide		
	*df*	*F*	*P*
**Habitat use**			
Rock	2.1378	6.15	**0.0022**
Algae + soft coral	2.1388	1.22	0.2953
Non-branching coral	2.1400	2.68	0.0691
Branching coral	2.1395	1.39	0.2493
Sand + coral rubble	2.1403	0.45	0.6390

Each habitat variable was ln-transformed prior to analysis. Snake ID was included as a random effect in all five models. Bold font indicates significant values (*P* < 0.05).

### Effect of tide on behaviour of snakes

**Figure 3:**
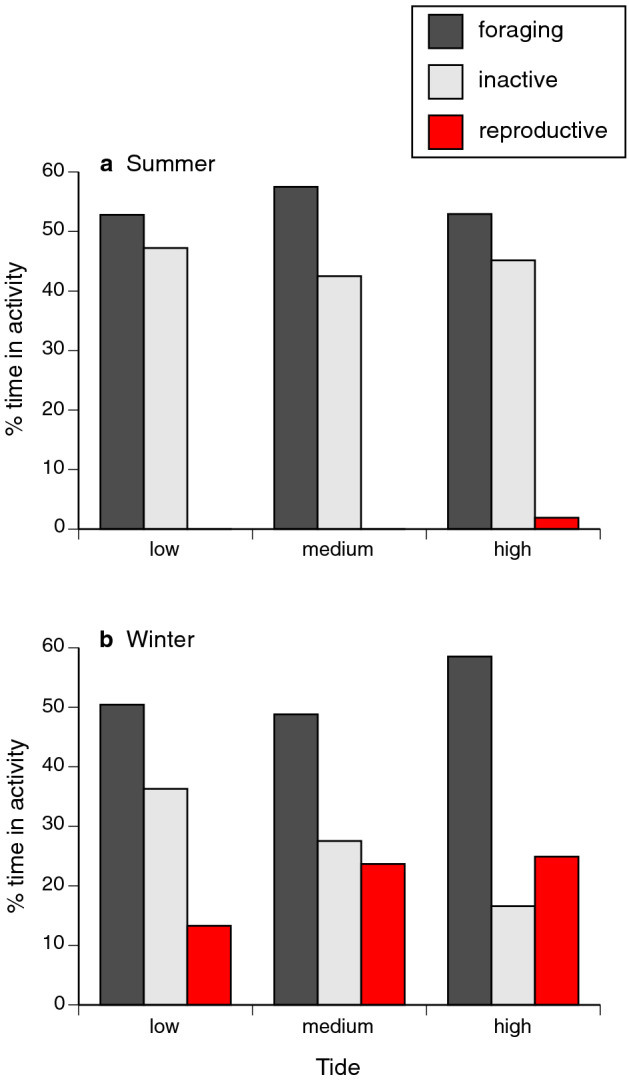
Interactive effects of tidal phase and season on the activity of sea snakes (*Emydocephalus annulatus*) in the Baie des Citrons, New Caledonia.

**Figure 4:**
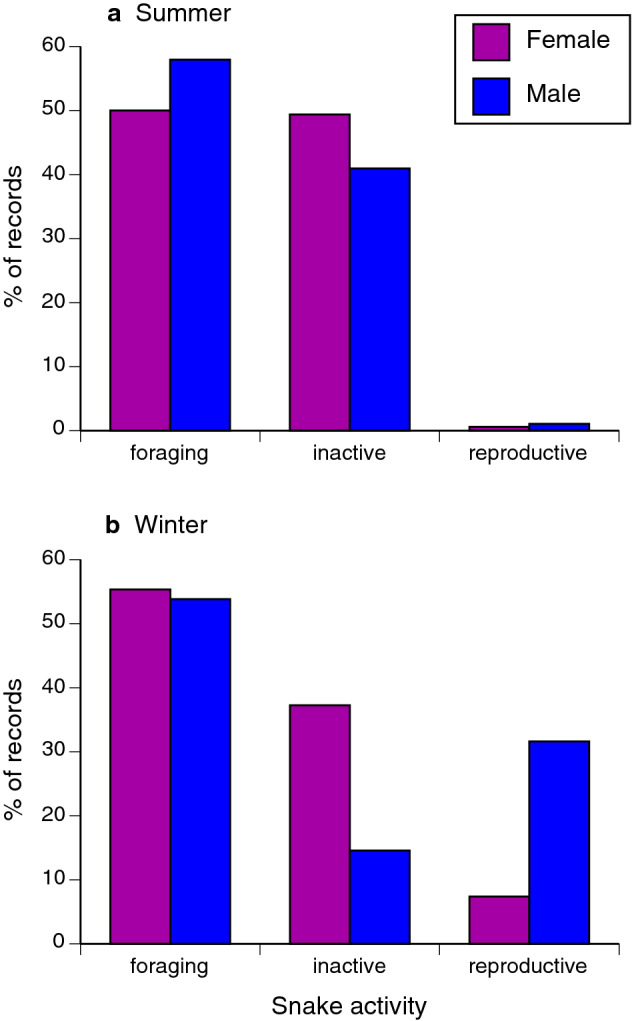
Interactive effects of season and snake sex on the activity of sea snakes (*Emydocephalus annulatus*) in the Baie des Citrons, New Caledonia.

Because reproductive behaviour differs between the sexes and is seasonal, we incorporated these factors into our analysis on the effect of tide on behaviour. The significant Tide*Season interaction (*F*_4,1168_ = 3.61, *P* = 0.0062; Table 2) revealed by this analysis indicates that the effect of tide on activity differed between summer and winter. Snakes were less active with decreasing tide height during winter but not summer (Fig. 3). Reproductive activity increased with tide height during winter, but this activity was largely absent during summer (Fig. 3). Activity of snakes was also significantly affected by the interaction between sex and season (*F*_2,1168_ = 6.11, *P* = 0.0023; Table 2). During winter, females were inactive more often than were males, and males were observed in reproductive activity more often than were females (Fig. 4). During summer, no sexual difference in these behaviours was apparent (Fig. 4).

**Table 2:** Effects of tide, sex and season on activity of sea snakes (*Emydocephalus annulatus*).

Effect	*df*	*F*	*P*
Tide	4.1168	3.00	**0.0178**
Sex	2.1168	5.84	**0.0030**
Season	2.1168	4.95	**0.0072**
Tide * sex	4.1168	1.31	0.2649
Tide * season	4.1168	3.61	**0.0062**
Season * sex	4.1168	6.11	**0.0023**

Three activity categories (inactive, foraging, and reproductive) were used as a three-factor multinomial dependent variable in a mixed model with snake ID as a random effect. Figures 3 and 4 show the probabilities of different activity categories predicted from this model. Bold font indicates significant values (*P* < 0.05).

## Discussion

The conclusion that tidal cycles influence activity patterns in sea snakes is unsurprising, but has remained speculative because of a lack of detailed behavioural data on these animals. For a population of sea snakes in a nearshore area with shallow water (as is true of our own study site), a decrease in water depth at low tide might induce two types of responses: the snakes might retreat to deeper water, or they might become inactive. Our data show that both of these responses occur. The sites where we find snakes at low tide are ones where water levels are high at peak tides (i.e. areas far from the shore), as expected if snakes retreat from the exposed shallows as water recedes with the tidal cycle. The same pattern could be generated by sampling artefacts, whereby snakes stay in the same site but we cannot see them at low tide because the water is too shallow for snorkelling. That alternative interpretation is falsified by the fact that many places where we see snakes at high tide are out of water at low tide. Snakes would be very obvious out of water, and we never see them in that situation. Tidal phase also affects activity, with snakes inactive at low tides during winter, foregoing reproductive activities (mate-searching and courtship) at that time. Foraging activity was relatively unaffected by tidal phase, with snakes shifting their feeding sites rather than abandoning foraging.

A trend for reproductive activity in males to increase with higher water levels in winter (the mating season^24^) likely reflects the ease of mate-searching in deeper water (because of a greater field of view), and a higher foraging level by females that renders them active, and thus easier to locate than when they are inactive at low tide. Experimental studies on mate-recognition in this population suggest that males experience great difficulty in locating and recognising females, due to a lack of the substrate-deposited pheromonal cues that guide mate-searching in terrestrial snake species^25^. As a result, males may abandon reproductive behaviours at low tide, when conditions are unfavourable for locating potential mates.

Tidal cycles may affect other species of sea snakes in different ways than in our own study. In some marine systems, a tidally-induced shift in proximity to the shoreline would necessarily modify the habitats in which snakes are found. For example, strict depth-associated zonation in substrate types would mean that snakes could exploit habitat types at high tide that were unavailable at low tide. The virtual lack of a tidally-enforced habitat shift at Baie des Citrons reflects the mosaic nature of the substrate, with the relative availability of different habitat types relatively independent of distance to shore. Similarly, because the fish eggs consumed by *Emydocephalus annulatus* are available at a wide range of water depths^24,26^, snakes are able to forage under all tidal conditions. Again, it is easy to imagine circumstances where this would not be true. Most sea snakes feed on fishes rather than fish eggs, and water depth or current flow may affect abundance of the snakes’ prey (or their ease of detection or capture). For such a species, foraging may be substantially affected by the tide. This is the case for *Hydrophis cyanocinctus*, which forage at low tide for mudskippers trapped within their burrows by the falling water levels^27^. A trend for *Hydrophis curtus* to move into areas where and when tidal fluctuations in water levels are maximal^16,17^ might well reflect availability of otherwise-shallow inshore areas that provide feeding opportunities. Likewise, snakes that are vulnerable to predation by large sharks at high tides might manifest strong habitat shifts with rising water levels^21,22^. Predation on *E. annulatus* at our study site appears to be minimal^28^ and thus, is unlikely to be affected by tidal phase. The extreme philopatry of Turtle-headed sea snakes^24,29,30^ means that an individual experiences strong tidally-driven shifts in water depths (because the snake does not move far in the course of a day), whereas individuals of a more vagile species could buffer such effects by moving over a greater area. Lastly, hydrodynamic challenges imposed by the strong currents sometimes associated with tidal cycles might curtail snake activity under those conditions^31^ (for *Hydrophis zweifeli*). Currents are minimal in the protected bay where we worked, minimising the importance of such challenges.

Widespread declines in populations of sea snakes have been reported, but the causes remain unclear. For example, several species of marine snakes have disappeared from sites that are protected from anthropogenic disturbance^32^. The same is true for the species that we studied, *E. annulatus*^33^. In other cases, pollution or coral bleaching may have catastrophic consequences for coral-reef ecosystems^34,35^. An understanding of the spatial ecology of sea snakes—including fundamental issues such as the impact of tidal cycles on snake behaviour and habitat use—may help to clarify these enigmatic disappearances. Many putative threatening processes differentially impact specific habitat types; for example, coral bleaching is concentrated in shallow areas, and affects particular types of coral^36^. Without a clear understanding of where and when sea snakes are active, and the abiotic and biotic factors that drive those patterns, it will be very difficult to tease apart potential causation for cases of population collapse. Understanding the drivers of habitat utilisation has proved to be helpful in addressing conservation issues in terrestrial snakes^14,37^, but our lack of knowledge about marine snakes curtails our ability to extend those insights to oceanic systems.

## Methods

### Species and study site

**Figure 5:**
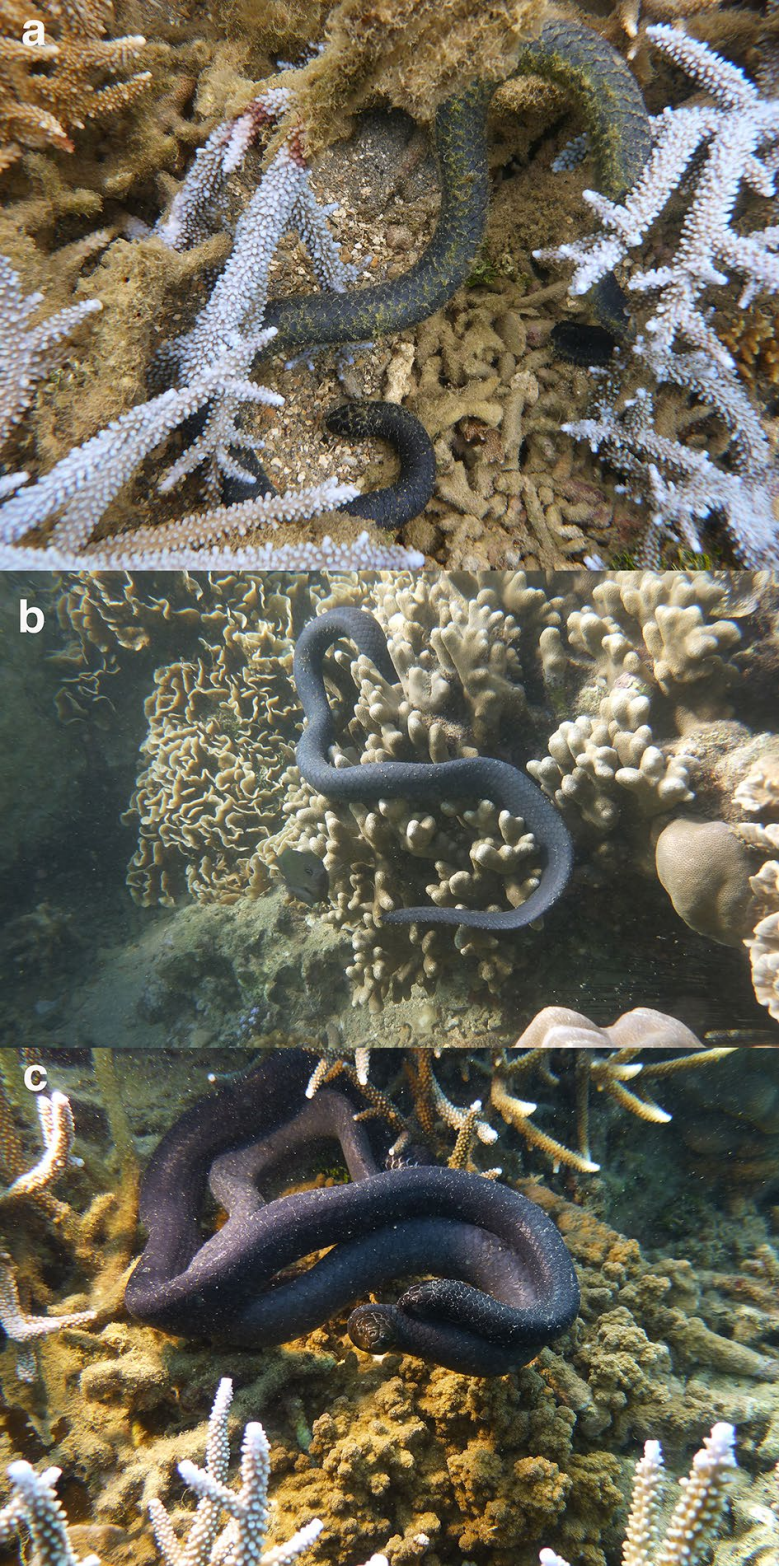
Turtle-headed sea snakes (*Emydocephalus annulatus*) in the Baie des Citrons, New Caledonia. The photographs show snakes (**a**) inactive, (**b**) feeding, and (**c**) courting. Photographs by Claire Goiran.

The turtle-headed sea snake (*E. annulatus*) is a thickset, medium-sized hydrophiine elapid^38^ (Fig. 5). In our study population, adult females average 61.9 cm snout-vent length (SVL); males are smaller (average 56.2 cm SVL). Some individuals are brightly banded in black and white, but most are melanic^29,39^. These snakes feed entirely on the eggs of demersal-spawning fishes, and are highly philopatric^24,29,30^.

We study turtle-headed sea snakes in the Baie des Citrons (“Lemon Bay”) beside the city of Noumea, New Caledonia (22° 16′ S, 166° 26′ E). The study area is 250 m long and 50–100 m wide at high tide), with a substrate dominated by sand in deeper water, and by corals, coral rubble and rocks in the shallows^26^. The shore region exposed at low tide is dominated by gently sloping coral rubble, an open habitat in which snakes would be clearly visible if they were present as the tide receded. The tidal range is usually less than 1.6 m, and headlands protect the bay from trade winds. Most snakes within this population carry microchips (Trovan ID) for individual identification. Research was conducted under permit 3252-17/ARR/DENV (Province Sud, New Caledonia) and ethics approvals 2015/880 (University of Sydney Animal Research Authority) and 2019/042 (Macquarie University Animal Research Authority). All methods were performed in accordance with the relevant guidelines and regulations.

### Data collection

On 312 days between March 2014 and August 2018, one to 12 divers snorkelled through the area to hand-capture snakes. Tidal stage was recorded as low, medium or high (i.e., the duration of a single tidal cycle between successive peaks and troughs was divided into three equal periods). All animals were checked for implanted microchips (with a scanner inside a waterproof bag) and then released immediately. We recorded water depth in the site at the time of capture, and the location of the capture based on our prior mapping of the area into 20 × 20-m quadrats. Within each 20 × 20-m quadrat, we subjectively scored substrate types (percent cover of rock, coral rubble, branching coral, non-branching coral, algae plus soft coral) and water depth at high tide. Whenever we sighted a snake, we classified its behaviour into one of three categories: (1) inactive (unmoving, partly hidden under coral etc.); (2) foraging (swimming slowly near substrate, tongue-flicking holes; and scraping at fish eggs^40^); and (3) reproductive (i.e. includes mate-searching—males moving rapidly, often midwater^41^, and courtship—male courting female, and female being courted^25^; Fig. 5).

### Data analysis

Detailed studies in this population have shown no strong links between phenotypic traits of a snake (body size, colour phase, sex, reproductive state, pregnancy) and its habitat use (substrate type, water depth^26,42^), so we did not include these characteristics in the current analysis. Instead, we focused on the numbers of snakes recorded in different situations (water depths, habitat types) at different tides (high, medium, low). We included data only for adult snakes (males > 35 cm SVL, females > 40 cm SVL), because our sample size of juveniles was too low for analysis.

To assess whether tide influenced which habitat characteristics were predominantly used by snakes, we used generalised linear mixed models implemented with proc glimmix in SAS 9.4 (SAS Institute, Cary, NC). Five models were run, with each of the five habitat types (percent cover of rock, sand plus coral rubble, branching coral, non-branching coral, algae plus soft coral) as dependent variables. Tide level (high, medium, low) was used as an independent variable in each model and individual snake ID was used as a random effect to account for pseudoreplication (multiple captures of the same individuals). These models were fit using normal distributions with identity link functions. Habitat variables were ln(1 + X)-transformed prior to analyses to improve normality.

We also used generalised linear mixed models to assess the effects of tide, sex and season (summer or winter) on behaviours of sea snakes. We analysed behaviour as a nominal dependent variable consisting of three levels (inactive, reproductive [courtship + mate-searching], foraging). The model contained Tide, Sex, Season and their interactions as independent variables, with snake ID as a random effect. In an initial model, the three-way interaction Tide * Sex * Season was nonsignificant (*F*_4,1164_ = 0.21, *P* = 0.93). Therefore, we ran a reduced model containing the main effects and all two-way interactions. Activity was modelled using a multinomial distribution, a generalised logit link function and an autoregressive error structure.

## Data Availability

The datasets generated and/or analysed during the current study will be deposited in the Dryad repository upon acceptance.
